# IRF-1 responsiveness to immune cytokines predicts different cancer phenotypes

**DOI:** 10.1186/2051-1426-1-S1-P249

**Published:** 2013-11-07

**Authors:** Daniela Murtas, Dragan Maric, Valeria De Giorgi, Jennifer Reinboth, Andrea Worschech, Patricia Fetsch, Armando Filie, Maria Libera Ascierto, Davide Bedognetti, Qiuzhen Liu, Lorenzo Uccellini, Lotfi Chouchane, Ena Wang, Francesco M Marincola, Sara Tomei

**Affiliations:** 1 Biomedical Sciences, Section of Cytomorphology, University of Cagliari, Cagliari, Italy; 2 Flow Cytometry Core Facility, NINDS, NIH, Bethesda, MD, USA; 3 Transfusion Medicine, CC, NIH, Bethesda, MD, USA; 4 Genetic Medicine, Weill Cornell Medical College in Qatar, Doha, Qatar; 5 Laboratory of Pathology, NCI, NIH, Bethesda, MD, USA; 6 Sidra Medical and Research Center, Doha, Qatar

## 

A dichotomy between immunologically active and quiescent cancer phenotypes has been demonstrated for several types of cancer. Central to such dichotomy is the master regulator of the acute inflammatory process interferon regulatory factor (IRF)-1. Although the relevance of IRF-1 to the immune biology of cancer is emerging, it remains unknown whether the responsiveness of IRF-1 to immune cytokines is able to point out different cancer behaviours. Here, we explored the significance of the IFN-γ- and TNF-α-induced activation of IRF-1 in 15 melanoma cell lines. We measured IRF-1 activation in 15 melanoma cell lines at basal level and after treatment with IFN-γ, TNF-α and a combination of both using ImageStream technique (Amnis Corp). Microarray analysis was applied to compare transcriptional patterns between cell lines characterised by high and low IRF-1 activation. Functional interpretation analysis was performed using the Ingenuity Pathway Analysis system (IPA) tools 3.0. We observed a strong positive correlation between IRF-1 activation at basal level and after IFN-γ and TNF-α treatment (ρ=0.65, p=0.007 and ρ=0.66, p=0.007, respectively) suggesting that both the cytokines have a strong effect in inducing IRF-1 activation. Given that different cell lines have different amplitude of response to cytokine stimulation, we next asked whether the inducibility of IRF-1 by IFN-γ and TNF-α was able to distinguish a parallel different behaviour of cancer cells. Toward this aim, we measured the difference of nuclear translocation score between controls and stimulated cells and further selected 3 cell lines with low and 3 cell lines with high delta IRF-1 nuclear translocation. Microarray demonstrated that the 3 cell lines with low and the 3 with high IRF-1 inducible translocation scores differed in the expression of 597 annotated transcripts. Functional interpretation analysis showed mTOR and Wnt/β-cathenin as the top upregulated pathways in the cell lines with low inducible IRF-1 activation, suggesting that a low IRF-1 inducibility recapitulates a cancer phenotype already described in literature characterised by poor prognosis. In conclusion, our findings suggest that the responsiveness of IRF-1 to IFN-γ and TNF-α is able to point out different cancer phenotypes and support the central role of this transcription factor in influencing different tumor behaviours.

